# Editorial: Decoding the Fetal Circadian System and Its Role in Adult Sickness and Health: Melatonin, a Dark History

**DOI:** 10.3389/fendo.2020.00380

**Published:** 2020-07-16

**Authors:** Claudia Torres-Farfan, Jose Cipolla Neto, Erik D. Herzog

**Affiliations:** ^1^Laboratory of Developmental Chronobiology, Faculty of Medicine, Universidad Austral de Chile, Valdivia, Chile; ^2^Centro Interdisciplinario de Estudios del Sistema Nervioso (CISNe), Faculty of Medicine, Universidad Austral de Chile, Valdivia, Chile; ^3^Department of Physiology and Biophysics, Institute of Biomedical Sciences, University of São Paulo, São Paulo, Brazil; ^4^Department of Biology, Washington University, St. Louis, MO, United States

**Keywords:** circadian rhythms, melatonin, DOHaD (developmental origins of health and disease), pregnancy, epigenetic

Throughout gestation, the newborn follows a precise development, preparing for a successful transition to extrauterine life. Maternal circadian signals not only play a key role in the precise daily delivery of oxygen, nutrients, hormones, and other biophysical signals but also synchronize daily rhythms in the fetus to the external photoperiod. As a result of changes in fetal demands during pregnancy, the maternal cardiac, respiratory, immune, renal, hepatic, and gastrointestinal, and endocrine systems differ from those of non-pregnant women (1, 2). The circadian system is not an exception; pregnancy-induced functional reorganization of daily rhythms in physiology and behavior has been reported in several species (3–5). Indeed, Martin-Fairey et al. (6) recently reported that pregnancy induces an earlier chronotype of activity in mice and women, with decreases in total activity close to delivery. This suggests that the maternal suprachiasmatic nucleus (SCN) and/or other components of the circadian system are modified during gestation. Consequently, this current special issue explores our current understanding about the role played by the circadian system in maternal well-being, fetal development, and diseases in adult life [(6); McCarthy et al.; van Dalum et al.; Beñaldo et al.; Mendez et al.].

As explained by McCarthy et al., melatonin serves as a key hormone signal from mother to fetus in several species, including humans. Expanding on this, articles in this issue by the groups of England and Herzog review how melatonin or other daily maternal signals may contribute to fetal development and the timing of birth and how circadian disruption may lead to adverse birth outcomes including preterm delivery [(6); McCarthy et al.; Bates and Herzog]. van Dalum et al. highlight how maternal photoperiodic programming, through a well-defined action of melatonin on the pars tuberalis, may provide an experimental paradigm to investigate the mechanisms by which early life experience establishes long-term patterns of hypothalamic regulation. For example, a recent report found that melatonin suppression during gestation resulted in decreased hepatic transcript levels of four enzymes involved in DNA methylation; maternal melatonin treatment reversed the effect in three of these enzymes. These results indicate that maternal melatonin modifies the epigenome in the fetal liver and opens the possibility of effects in other fetal organs (7).

Accordingly, Torres-Farfan et al. (7) and Cipolla-Neto and Amaral (8) provide insight into how melatonin treatment may be a therapeutic against circadian misalignment. Importantly, special attention should be taken about the precise timing of melatonin treatment. For example, the recent studies of Beñaldo et al. in sheep found that melatonin treatment when the newborn did not produce melatonin induced negative effects in cardiovascular function and in response to chronic hypoxia.

Finally, Mendez et al. report here that the rat offspring from mothers exposed to chronic photoperiod shifts suffer several alterations in the maturation of renal and immune function, consistent with adult hypertension (9). Taken together, the articles in this special issue emphasize how irregular daily schedules during pregnancy can impact maternal–fetal communication and fetal development and put the offspring at risk for adult disorders including hypertension, type II diabetes, and obesity.

## Author Contributions

All authors listed have made a substantial, direct and intellectual contribution to the work, and approved it for publication.

## Conflict of Interest

The authors declare that the research was conducted in the absence of any commercial or financial relationships that could be construed as a potential conflict of interest.
